# Supplementary material for: Exploring the relationship between the food environment and preferences among schoolchildren in a low socioeconomic community in Sri Lanka: A GIS-based assessment
Source: PLoS One. 2024 Aug 13;19(8):e0291893. doi: 10.1371/journal.pone.0291893 (PMC11321566; doi:10.1371/journal.pone.0291893)
Supplement: S1 File — (PDF) [file pone.0291893.s001.pdf]

Dear Sir/Mdam,

Submitting additional documents in relation to ethical approval of the research (PONE-D-23-28601)

I am writing to provide additional documentation pertaining to the ethical approval of my manuscript with the reference number PONE-D-23-28601. This supplement is in response to your request for supplementary material concerning the ethical aspect of my research titled "Exploring the association between neighborhood school environment and preferences of school children in a low-come community: A GIS based assessment".

First, I would like to highlight that this manuscript is an integral part of my postgraduate degree (MPhil) and I have enclosed supportive documents in appendix 01. The data collection for the portion of this research that is pertinent to the current paper tool place during late 2016 and 2017. It is essential to note that during this research period I obtained two extensions, the first of which was granted in 2015 (as documented in appendix 02), and the second extension was approved in 2017. It is important to mention that during my second extension request, I also sought an amendment to the methodology section. However, Ethics Review Committee (ERC) approved this amendment as indicated in the letter enclosed in appendix 03, and letter does not specify the extension period. Additionally, I am providing several documents that serve to demonstrate the ongoing communication between the principle investigator and the ERC. Also I requested a copy of the extended approval once I recived your request for further documentation. Regrettably, ERC confirmed that my file had been disposed of, as I had submitted my final report in 2018, as reflected in the email communication found in appendix 05.

Based on the above evidence, I firmly believe that these document substantiate the communication and interaction I had with the ERC, which were vital in ensuring the ethical integrity of my research. I kindly request your consideration for this evidence and your assistance in advancing the manuscript to the assessment stage.

Thank you for your attention and support in this matter

Chamil Senevirathne

Appendix 01

RESEARCH & HIGHER DEGREES COMMITTEE

Faculty of Medicine
University of Colombo

P.O. Box 271, Kynsey Road
Colombo 8
SRI LANKA
Tel : 94 011 2695300
Fax: 94 011 2691581

15th March 2019

Mr. C P Senavirathne
No. 14, 'Madara'
Walala Junction,
Walala,
Menikhinna.

Dear Mr. Senavirathne,

Title: "Assessment of school food environment and its relationship with the nutritional status among children in the District of Moneragala" 2014-14.

Thank you for submitting the above titled progress reports for the period between January 2018 and June 2018 AND July 2018 and December 2018, which was approved by the RHDC and recommended at the Faculty Board at its 459th meeting held on 12th March 2019.

Thanking you,

Yours sincerely,

Ms. Lakshmi Jayalath
Secretary / Research and Higher Degrees Committee

Secretary
RHDC
Faculty of Medicine
University of Colombo

Contact details

Chairperson:
Professor Vajira H.W Dissanayake
Department of Anatomy
Tel. 2689135

Secretary:
Ms. Lakshmi Jayalath
Department of Allied Health Sciences
Tel. 2688686

Appendix 02

Ethics Review Committee

Faculty of Medicine
University of Colombo
P O Box 271, Kynsey Road, Colombo 8, Sri Lanka
Telephone: +94-11-2695300 ext 240 Fax: +94-11-2691581
Email: ethicscommitteemfc@gmail.com

REFERENCE: EC-14-105

13th November 2015.

Dr Prasad Katulanda,
Department of Clinical Medicine,
Faculty of Medicine,
Colombo.

Dear Dr.Katulanda,

RE	:	**Protocol No EC-14-105**
Title	:	**Do surrounding school environment promotes nutrition of children: a GIS mapping based assessment of the impact of school neighborhood on the nutritional status and practices among school children in the District of Moneragala**
Investigators	:	**Dr Prasad Katulanda**<br>**Mr. Chamil Senevirathne**<br>**Dr Kremlin Wickramasinghe**<br>**Dr. Padmal de Silva**

The request made by you to extend the ethics approval period from , 18.09.2015 in order to complete your research was considered by the the Executive Committee of the Ethics Review Committee, at its meeting on 13.11.2015 and approval was granted for this extension.

Ethics Review Committee
Faculty of Medicine
University of Colombo
P O Box 271, Kynsey Road, Colombo 8, Sri Lanka
Telephone: +94-11-2695300 ext 240 Fax: +94-11-2691581
Email: ethicscommitteemfc@gmail.com

You are asked to note the following:

This approval is valid for one year, and the Committee requires that you furnish it with a final report.

- This approval relates to the ethical content of the study only, and you are responsible for the following:
  - negotiating individual arrangements with the Heads of service departments in those situations where the use of their resources is involved,
  - if appropriate, informing the study sponsor that the membership and procedures of the University of Colombo, Ethics Review Committee comply with the relevant guidelines of the Forum of Ethics Review Committees in Sri Lanka.

Yours sincerely,

Prof. Hemantha Senanayake
Chairman
Ethics Review Committee
Faculty of Medicine
University of Colombo

Ethics Review Committee
Faculty of Medicine
University of Colombo
Kynsey Road
Colombo 8

Appendix 03

Ethics Review Committee
Faculty of Medicine
University of Colombo
P O Box 271, Kynsey Road, Colombo 8, Sri Lanka
Telephone: +94-11-2695300 ext 240 Fax: +94-11-2691581
Email: ethicscommitteemfc@gmail.com

REFERENCE: EC-14-105

8th December 2015.

Dr Prasad Katulanda,
Department of Clinical Medicine,
Faculty of Medicine,
Colombo.

Dear Dr.Katulanda,

RE : **Protocol No EC-14-105**

Title : **Do surrounding school environment promotes nutrition of children: a GIS mapping based assessment of the impact of school neighborhood on the nutritional status and practices among school children in the District of Moneragala**

The request made by you to approve the following amendment to the above study was considered by the Executive Committee of the Ethics Review Committee, at its meeting on 02.12.2015 and approval was granted:

i) to change the title of the study as " Assessment of school food environment and its relationship with the nutritional status among children in the district of Monerabgala (As per the front page of the title changed application)
ii) to change the general and specific objectives of the study **(As per Version 1.1 proposal)**
iii) to use a food availability check list to determine the availability of healthy foods and their prices. **(As per Version 1.1 proposal & version 3.1 Data Collection form)**
iv) to remove the previous " Food stall assessment" check list and to use the similar food availability check list which is to be used to assess the school canteens **(As per Version 1.1 proposal & version 3.1 Data Collection form)**

Yours sincerely,

Prof. Hemantha Senanayake
Chairman/ Ethics Review Committee
Faculty of Medicine
University of Colombo

Ethics Review Committee
Faculty of Medicine
University of Colombo
Kynsey Road
Colombo 8

Appendix 04

Ethics Review Committee

Faculty of Medicine
University of Colombo
P O Box 271, Kynsey Road, Colombo 8, Sri Lanka
Telephone: +94-11-2695300 ext 240 Fax: +94-11-2691581
Email: ethicscommitteemfc@gmail.com

REFERENCE: EC-14-105

23rd August 2017.

Dr Prasad Katulanda,
Department of Clinical Medicine,
Faculty of Medicine,
Colombo.

Dear Dr.Katulanda,

RE : **Protocol No EC-14-105**

Title : **Assessment of school food environment and its relationship with the nutritional status among children in the district of Moneragala**

The request made by you to approve the following amendment to the above study was considered by the Executive Committee of the Ethics Review Committee, at its meeting on 22.08.2017 and approval was granted:

i) to increase the number of clusters by 10 clusters to the study to recruit the required number of students from selected schools. (As per Version 1.2 proposal)

Yours sincerely,

Dr.Enoka Corea
Chairperson
Ethics Review Committee
Faculty of Medicine
University of Colombo

Ethics Review Committee
Faculty of Medicine
University of Colombo
Kynsey Road
Colombo 8

Appendix 05

Requesting a copy of extension letter - EC.14.105 2 Yahoo/Inbox

chamil senaviratne <cpchamil@yahoo.com> Fri, 20 Oct at 7:59 am

To: ethicscommitteemfc@gmail.com

Dear Sir/Madam,

I am writing this to request the ethical approval extension letter for the application EC.14.105.

As I submitted a manuscript to the journal PLOSONE, they have requested submit a copy of extension granted letter upon the above application. If the file is still available, could you please provide a copy of that letter. This would be a great help.

Thank you !

Chamil Priyanka Senvirathna
Lecturer
Department of Health Promotion
Faculty of Applied Sciences
Rajarata University of Sri Lanka.

Mobile +94711587459
Office +94252266131
Linkdin : Chamil Senevirathna
Orchid id : cpchamil

ethics committee <ethicscommitteemfc@gmail.com> Fri, 20 Oct at 11:05 am

ethics committee <ethicscommitteemfc@gmail.com> Fri, 20 Oct at 11:05 am

To: chamil senaviratne

Dear Sir/Madam,

We are sorry. We removed the file when you submitted the final report earlier. Final report is attached herewith for your reference

Thanks and regards
Piumali

Show original message

--
Ethics Review Committee
Faculty of Medicine
University of Colombo
Sri Lanka

EC-14-105 ... .docx
15.7kB

To be completed in typescript and submitted by the Principal Investigator (PI).

1. Details of PI

Name:	Dr. Prasad Katulanda
Address:	Senior Lecturer, Department of Clinical Medicine, Faculty of Medicine, University of Colombo
Telephone:	0772929991
E-mail:	prasad.katulanda@yahoo.com

2. Details of study

Full title of study:	Assessment of school food environment and its relationship between the nutritional status among the school children in the district of Moneragala, Sri Lanka
Protocol number:	EC-14-105
Date of ERC approval:	16.09.2014
Is this is an Annual Progress Report or a Final Report	Final Report
Duration for which the report is provided (Reporting Period)	14.07.2017 to 09.06.2018

3. Progress to date

The research is completed and draft of the thesis is shared with supervisors for their comment. Final thesis to be submitted in another one month of time.

4. Ethical Issues

Maintenance and security of records	All the study subjects were identified by a code (Student ID). Data were collected and transferred to a data base. Measures have been taken to secure data and all the questionnaires are safely stored for future purposes.
Compliance with approved protocol	Data were collected from school children, school canteen owners and neighboured shop owners. Data collection was done according to the approved protocol. Information sheets were given and written consent was obtain from each subject prior to the data collection.
Any changes to the protocol including any planned for the next year	Few changes were made and ethical review committee was informed and approved.
