# Supplementary material for: Exploring the relationship between the food environment and preferences among schoolchildren in a low socioeconomic community in Sri Lanka: A GIS-based assessment
Source: PLoS One. 2024 Aug 13;19(8):e0291893. doi: 10.1371/journal.pone.0291893 (PMC11321566; doi:10.1371/journal.pone.0291893)
Supplement: Attachment — Submitted filename: Rebuttal letter_PONE-D-23-28601.pdf [file pone.0291893.s003.pdf]

C.P Senevirathne
Department of Health Promotion
Faculty of Applied Sciences,
Rajarata University of Sri Lanka
31.01.2024

Dr. Emily Chenette
Editor-in-Chief
PLOSONE

Subject: Rebuttal to Reviewer Comments on Manuscript PONE-D-23-28601

Dear Dr. Chenette,

I hope this letter finds you well. I appreciate the time and effort invested by the reviewers and the editorial team in evaluating my manuscript titled **“Exploring the Relationship between Food Environment and Preferences of School Children in a Low-Socioeconomic Community in Sri Lanka: A GIS-based Assessment**. I am grateful for the constructive feedback provided, and I have carefully considered each comment in the revision of my manuscript. We found that each comment provided by both reviewers is highly valuable, and contributes to ensuring the quality of the manuscript.

The summary of the reviewer’s comments and clear and concise responses to the first reviewer's comments have been addressed, and specific changes are incorporated into the revised manuscript which are mentioned in highlighted areas of the manuscript, and as given in the summary table below.

Additionally, a few modifications in the title page, methodology, ethical statement, and references section were made to ensure the meeting of PLOS ONE journal criteria. Further, measures were taken to improve the language of the content.

Therefore, I assure you that the revised version of the manuscript fully adheres to the journal’s guidelines and policies.

Thank you for considering my manuscript for publication in PLOS ONE. I appreciate your time and consideration.

Sincerely,

Chamil Senevirathne

chamil@as.rjt.ac.lk +94711587459

Summary table of responding to reviewer's comments – PONE-D-23- 28601

Addressing the comments given by reviewer -01		
01	In the introduction, the authors seek to explain the dimensions of the FAO's definition of the school food environment, however, they do not address food prices. I recommend going through this dimension, even if the focus is on availability, as it was discussed about the nutritional content of foods and marketing.	I agree with the comment and amended the introduction section accordingly<br><br>Introduction section Page:
02	The maps created in Figure 1 are ineligible. It is essential to improve the resolution of the images.	While agreeing to this comment, I changed the map by improving the resolution.<br><br>Result section Page: 13

Addressing the comments given by the reviewer- 02		
01	Major Comments	Correction
01	I suggest revising the titles of all figures and tables to include details about the population group, location, year of data collection, and sample size (n =).<br>Lines 133-134: I understand that "nutrition practices" refer to the overall consumption of selected healthy and unhealthy eating markers, not specifically when purchased in school vicinities. However, there is a potential for misunderstanding when the authors present their conclusions (lines 324-327). To avoid confusion, despite the potential redundancy, I recommend that the authors explicitly clarify this in the methodology and throughout the text. They can use terms such as "general healthy eating markers" and emphasize that the information pertains to the last 30 days	According to this suggestion, we change all the figure and table titles<br><br>In our study, we referred to nutrition practices about food consumption from neighborhood food outlets. Hence, we agreed to use the term general healthy eating makers throughout the article. We modified the conclusion too.
02	Lines 131-137: Please, clarify whether the translation and pre-test of the Global School Health Survey (GSHS) questionnaire were part of the	We address this point on page number 06. This instrument is

	referenced validation study [20] or if these steps were conducted by the research team during the planning phase of this research.	already validated by the Ministry of Health In Sri Lanka.
03	Lines 141-142: The authors should provide a general statement elucidating the conceptualization of healthiness adopted in the school canteen policy. For instance, specifying criteria such as energy-dense foods, items with added sugar, high-fat foods, and ultra-processed foods would enhance clarity.	This section is further elaborated on page 07. In addition, on page 04, in the introduction section, we have discussed the implications and their challenges.
04	Lines 142-143: In my view, stating that "Food environment was defined as all the outlets selling food within 300 meters from the school" may be misleading. The term "food environment" is a construct with established definitions in the literature. It seems you intended to convey that they defined the school vicinities within this radius, which would be a more accurate description.	First, we defined the term food environment using recent literature, which is included in the introduction section. Further, we defined a 300-meter buffer zone for our study<br>Methodology section<br>Page: 08
05	Lines 142-146: Please provide additional details to ensure that readers fully comprehend how you determined the 300m radius. Did you select a subset of students to investigate the average distance between their homes and the school? If so, could you elaborate on the methodology employed?	This comment was addressed while explaining the sections about the comments 06 and 07<br>Methodology section<br>Page -08
06	Lines 156-157: Clarify the variables to which you are referring in the statement "...association between the variables and the dietary choices...". Specify the particular variables involved in this analysis.	All the dependent and independent variables of the chi-square and regression analysis were explained.<br>Methodology section<br>Page- 08
07	Lines 205 and 208: Upon reviewing your methods, it appears that you did not inquire students about specific foods they purchase in the school neighborhood. However, the term "snack" is used here. In the methods section, clearly specify the nature of the questions asked, and subsequently, revise the results and discussion sections to eliminate any ambiguities. For instance, in line 208, where you report results about dietary trends, it seems to refer to the consumption of eating markers in the last 30 days, regardless of the place of purchase and consumption. However, in Table 5, the outcome variable is labeled "Consuming energy-dense food from outlets," causing confusion again.	In this analysis, we collected information on different types of food that study participants. As you mentioned in the comment, truly it did not reflect from the method section. Hence, we have

		modified the explanation in the method section.<br><br>In addition, I have with this useful comment, I have elaborated on the process of collecting GIS data in the same section.<br>Methodology section<br>Page : 08
08	Lines 201-212: I recommend moving these individual level results to line 192, right after Table 3 (including the Figure 2). Then you bring the food environment results and, in the last place, the associations between them.	I have re-arranged the order adhering to this comment.<br><br>Results section<br><br>Pages: 11 and 12
09	Table 5: Since you present odds ratios in your results, I assume logistic regression analyses were performed instead of chi-squared tests. However, this is not explicitly mentioned in your methods. Additionally, it might be more accurate to label the variable you named as "Availability of unhealthy food" as "Presence of establishments selling any unhealthy food."<br>If you possess information on the specific types of foods available for purchase in the food establishments and details about the consumption of these items by the students, I strongly recommend conducting further analyses to demonstrate these associations. This would provide a more nuanced understanding beyond the association between the presence of establishments selling ANY unhealthy food and the consumption of ANY unhealthy food.	I completely agree with this point and managed to modify the table and interpretation. Hence, I have specified the dependent variable added different food types, and calculated OR and correlation.<br><br>Results section<br>Pages 15-16
10	Table 6: It seems that you are referring to the consumption of ANY unhealthy food (yes vs. no). Readers might find it particularly valuable to observe relationships stratified by the types of food items available for purchase and those actually consumed. Moreover, I am unclear about the meaning of "Coefficient 95% CI." Where is the 95% confidence interval, and what does this coefficient represent? Is it a beta? You mentioned Spearman correlation analysis in the methods, but it seems incongruent in the context of these two variables. Furthermore, both association analyses presented in Tables 5 and 6 should be multivariable analyses instead of just bivariate analyses. There are potentially significant confounding variables at both the individual and school neighborhood levels (especially socioeconomic status), and it would be essential to adjust the analyses accordingly.	Considering this valuable comment, I have changed the analysis accordingly. I have expanded the multivariate regression analysis to observe the food consumption (by its different items) - thereby, I have modified the table 6, as well as the interpretation, and

		then modified the discussion section.<br>Results section<br>Pages : 16-17 and 18.
Minor comments		
01	Lines 73-74: Is the information about school types relevant in the context of the study? It seems to me that it doesn't add information.	Removed from the text<br>Introduction section<br>Page: 04
02	Line 97: I don't understand what is "economic status of the study".	This study is particular to the economic community in Sri Lanka. Hence, we used poverty indicators published by the Department of Census, to justify this fact. However, there is a typo that doesn't fully describe our point. So changed accordingly<br>Methodology section<br>Page: 05
03	I recommend formatting the tables according to international standardized patterns. Additionally, I suggest combining the information from Tables 1 and 2 into a single table, making the necessary changes in its title. Please ensure the titles of Figure 1 are included, and correct the numbering of Figure 2, which is currently labeled as 1.	Table 1 and 2 was amalgamated, and changed the description.<br><br>Adhering to this comment, all the tables were re-named and footnotes were included.<br>Results section<br>Pages 11. 13, 15, 16 and 18
04	Regarding Figure 2, showing snacks consumed by students, it does not seem to be referenced in the results section. Please address this inconsistency.	Changed the text to reflect the content of Figure 2<br>Results section<br>Page:14
05	For Table 4, include the results for the role of health and unhealthy food categories. Furthermore, add information about the statistical tests performed in the footnotes of the tables.<br>In the results section, avoid using statements such as "As presented in	Changed the whole content of Table 4.<br>Result section<br>Page: 16 & 17

	Table 2...". Instead, simply reference the titles of the tables and figures within parentheses	
06	A major language review is necessary to enhance readability throughout the manuscript.	The language was improved with the support of a few language experts

meeting the requirements of PLOS ONE

01	You may seek permission from the original copyright holder of Figure 1 to publish the content specifically under the CC BY 4.0 license.	.These maps were developed using the GIS analysis process. Hence, all the maps included in this manuscript have been generated by the authors.
