# Supplementary material for: Exploring the relationship between the food environment and preferences among schoolchildren in a low socioeconomic community in Sri Lanka: A GIS-based assessment
Source: PLoS One. 2024 Aug 13;19(8):e0291893. doi: 10.1371/journal.pone.0291893 (PMC11321566; doi:10.1371/journal.pone.0291893)
Supplement: Attachment — Submitted filename: Rebuttal letter_PONE-D-23-28601_Second review.docx [file pone.0291893.s004.docx]

C.P Senevirathne

Department of Health Promotion

Faculty of Applied Sciences,

Rajarata University of Sri Lanka

01.06.2024

Dr. Emily Chenette

Editor-in-Chief

PLOSONE

**Subject: Rebuttal to Reviewer Comments on Manuscript PONE-D-23-28601**

Dear Dr. Chenette,

I hope this letter finds you well. I appreciate the time and effort invested by the reviewers and the editorial team in evaluating my manuscript titled “Exploring the Relationship between Food Environment and Preferences of School Children in a Low-Socioeconomic Community in Sri Lanka: A GIS-based Assessment. I am grateful for the constructive feedback provided, and I have carefully considered each comment in the revision of my manuscript. We found that each comment provided by both reviewers is highly valuable, and contributes to ensuring the quality of the manuscript.

The summary of the reviewer’s comments and clear and concise responses to the reviewer's comments have been addressed, and specific changes are incorporated into the revised manuscript which are mentioned in highlighted areas of the manuscript, and as given in the summary table below.

Therefore, I assure you that the second revised version of the manuscript fully adheres to the journal’s guidelines and policies.

Thank you for considering my manuscript for publication in PLOS ONE. I appreciate your time and consideration.

Sincerely,

Chamil Senevirathne

[chamil@as.rjt.ac.lk](mailto:chamil@as.rjt.ac.lk) +94711587459

**Summary table of responding to reviewer’s comments – PONE-D-23- 28601**

| Addressing the comments given by reviewer -03 | | |
| --- | --- | --- |
| 01 | In the methods (Line 97), it is important to provide more information about the poverty indicator, including the data source - Department of Census - and a description of the variables included in this indicator. | Page No 05: Based on the updated report published by the Department of Census and Statistics in Sri Lanka, I have expanded this section to introduce further information on poverty indicators |
| 02 | The authors have presented frames instead of tables. I recommend revising the presentation and using the format of a table rather than a frame. | I have slightly modified the tables by removing frames. Now the tables are adhered to the PLOS one guidelines. |
| 03 | Although the maps have significantly improved in quality compared to the previous version, it is still difficult to see the points. I recommend increasing the size of the points on the maps to enhance visibility. | Page no 14, I have modified the maps improving the resolutions. |
